# Determination of Residential Soil Gas Radon Risk Indices Over the Lithological Units of a Southwestern Nigeria University

**DOI:** 10.1038/s41598-020-64217-8

**Published:** 2020-04-30

**Authors:** Deborah Tolulope Esan, Mynepalli Kameswara Chandra Sridhar, Rachel Obed, Yinka Ajiboye, Olusegun Afolabi, Babakayode Olubodun, Olatunde Michael Oni

**Affiliations:** 1 Department of Environmental Health Sciences, Faculty of Public Health, University of Ibadan, Ibadan, Nigeria and Department of Nursing Science, AfeBabalola University, Ado-Ekiti, Nigeria; 2 Department of Physics, University of Ibadan, Ibadan, Nigeria; 3 Department of Community Health, ObafemiAwolowo University, Ile-Ife, Nigeria; 4 Department of Geology, ObafemiAwolowo University, Ile-Ife, Nigeria; 5 Department of Pure and Applied Physics, LadokeAkintola University of Technology, Ogbomoso, Nigeria; 6 Department of Mathematical and Physical Sciences, AfeBabalola University, Ado-Ekiti, Nigeria

**Keywords:** Natural hazards, Oncology

## Abstract

Radiation dose from natural sources is mainly from exposure to radon in the environment. Radon has its origin from uranium-bearing bedrocks and overburden. In the present study, assessment of the level of radon over the three lithological units upon which the residential areas of ObafemiAwolowo University Campus, Ile-Ife (OAU) was situated was carried out. Soil gas radon concentration measurement was carried out at a constant depth of 0.80 m across the three lithologies (granite gneiss, grey gneiss and mica schist) using a RAD7 electronic radon detector. A total of 138 *in-situ* soil gas radon measurements were carried out. Obtained experimental data were analysed and summarised using descriptive and inferential statistics with statistical significance set at p < 0.05. A radon potential map was also developed using existing permeability data of the soils in the area. Soil radon concentration varied across the different lithologies ranging from 0.04 kBq/m^3^ – 190 kBq/m^3^ with a mean value of 14 kBq/m^3^. The mean value of Rn-222 concentration obtained in the three lithologies are 3.5 ± 5.9, 11.5 ± 25.8 and 28.4 ± 37.4 kBq/m^3^ for granite gneiss, grey gneiss and mica schist respectively. There is a statistically significant difference (p < 0.001) in the mean concentration of radon-222 measured on the three lithologies. The granite gneiss and grey gneiss lithologies have been designated into low radon index, while mica schist lithology has been designated as medium radon index. 34% of the sampled areas exhibit high radon risk based on Swedish risk criteria, thereby warranting protective actions.

## Introduction

Human exposure to radon (Rn-222) present in soil gas can have adverse effect on health. Radon is a unique radioisotope because it exists in gaseous form, unlike other radioactive elements. Since gases exhibit greater mobility than other states of matter, radon can diffuse from regions of higher concentration to regions of lower concentration. Radon is produced in uranium bearing rocks and soils. It is produced when radium (Ra-226), a radioactive daughter in the uranium (U-238) decay chain, decays by emitting alpha particle. Due to the different levels of uranium mineralization of soils in different places, radon concentration in soil varies from place to place in the world.

The radon gas produced in the upper layer of soil tends to move through soil pores to the surface due to the pressure difference between the surface air and the soil gas containing radon. The soil gas radon which has moved to the outdoor air pose insignificant hazard to human health since it is highly diluted by outdoor air. The soil gas can also move into buildings through channels such as sewage pipes, holes between concrete walls, spaces between floor tiles and raised floors, etc. If a building is poorly ventilated, the radon gas can accumulate to concentration level that is hazardous to health. Generally, the concentration of radon indoors is generally determined by a number of factors such as the type of heating, ventilation, and air conditioning (HVAC) system, building material, room dimension, building age, geomorphology and lithology. Although some buildings materials containing parent radionuclides of radon can also contribute to indoor radon concentration, however, soil gas containing radon found in soils overlying basement rocks constitute the main source for indoor radon concentration^1^. This contribution by soil gas radon to indoor radon concentration is majorly determined by the radon concentrations a few meters beneath the soil surface^2^. Also, the rate at which radon enters into indoor environment depends on the level of uranium mineralization of the underneath bedrock, gaseous pressure gradient in the soil, extent of fracture of the underneath bedrock, porosity of the overburden, soil moisture, permeability and temperature gradient^3,4^.

Several studies on indoor and outdoor radon have associated radon exposure to lung cancer risk^5–7^. Radiation received from natural sources is majorly from radon as it accounts for over 50% radiation dose received from natural sources^8^. After smoking, radon is responsible for the incidences of lung cancer^6^. From many cancer surveys that have been carried out across the world, lung cancer incidence constitutes a range proportion between 3% and 14% of the entire cancer incidences^9^. When radon gas is inhaled, even though most of it is exhaled, some of the radon atoms have the tendency to decay along the respiratory tract. In the process, the decay products of radon, which includes ^218^Po, ^214^Po and others, which are solids, get attached to the respiratory tract. These decay products typically have short half-lives. They decay by emitting highly energetic alpha particle along the respiratory tract. The emitted alpha particles have the potential to initiate carcinogenesis. In a study conducted by Collier, *et al*.^10^ on rats exposed to radon, evidence of lung cancer was found among the exposed rats. The theory adduced for the carcinogenesis is damage to DNA caused by direct hit of alpha particles from polonium on the DNA or indirect damage caused by fast-moving radiation-induced free radicals in the cell^11^. Radon-induced cancer ranks high among other preventable causes of death. In the United States, it was reported that the average person gets more radiation dose from exposure to indoor radon than from any other source of natural or man-made radiation^12^. In another study of the indoor radon level in some Indian cities, Khan^13^ reported a lifetime cancer risk of about 0.3%, a probabilistic value which indicates the tendency of 3 persons from a population of 1000 developing lung cancer over a lifetime period. Also, in a study by Darby, *et al*.^14^ whichanalysed several case-control studies on residential radon exposure across Europe, it was found that exposure to indoor radon accounts for 2% of deaths from Cancer in Europe.

Characterization of soil gas radon in an environment based on superficial geology is a useful tool to determining indoor radon concentration^15,16^. Areas underlain with granitic rocks which are mostly enriched in uranium are usually radon prone^17,18^. Similarly, volcanic regions have been reported to have elevated indoor radon concentrations^3,19^. However, in comparison to soil gas radon concentration in areas underlain by granitic rocks, sedimentary formation areas usually exhibit lower radon concentration^20^. For radiological planning and mitigation purposes, geogenic mapping is essential to limit human exposure to radon and its consequent radiological hazard.

In line with the national action plan recommendations of the International Commission on Radiological Protection^21^, which suggested the use of radon map for optimization of search for homes or areas with high radon concentration in order for preventive action to be taken for construction of new buildings, this study employed spatial radon measurement for development of radon distribution map. Spatial radon distribution mapping is a very important predictive tool which has been used across the different countries of the world for implementation of protective and remedial measures against adverse health effect exposure to indoor radon^17,22–24^. The method generally involves *in-situ* measurement of soil gas radon on regular grids on different geological units^17^. Literature survey shows there is sparse data on soil gas radon in Nigeria, and spatial radon mapping in Nigeria is also sparse. The sampled site is one of the foremost Universities in Nigeria and it has no spatial radon map which could be used for the purpose of planning mitigation of radon exposure and its potential radiological health hazard.

## Methods

The research employed an analytical cross-sectional design. The study was conducted in staff residential quarters of ObafemiAwolowo University Campus, Ile-Ife, Osun State. Ile-Ife is an ancient town of southwest Nigeria located on 7^o^28′′30′N latitude and 4^o^28′30′′E longitude. The elevation is between 220 and 330 meters above sea level. It lies within the tropical rain forest region of Nigeria. The area is characterised by two distinct seasons. The rainy season usually from April to October, and the dry season from November to March. The annual rainfall ranges between 150 to 3000 mm with varying relative humidity between 40% to 98%. The diurnal temperature varies from 23 °C to 39 °C.

**Figure 1:**
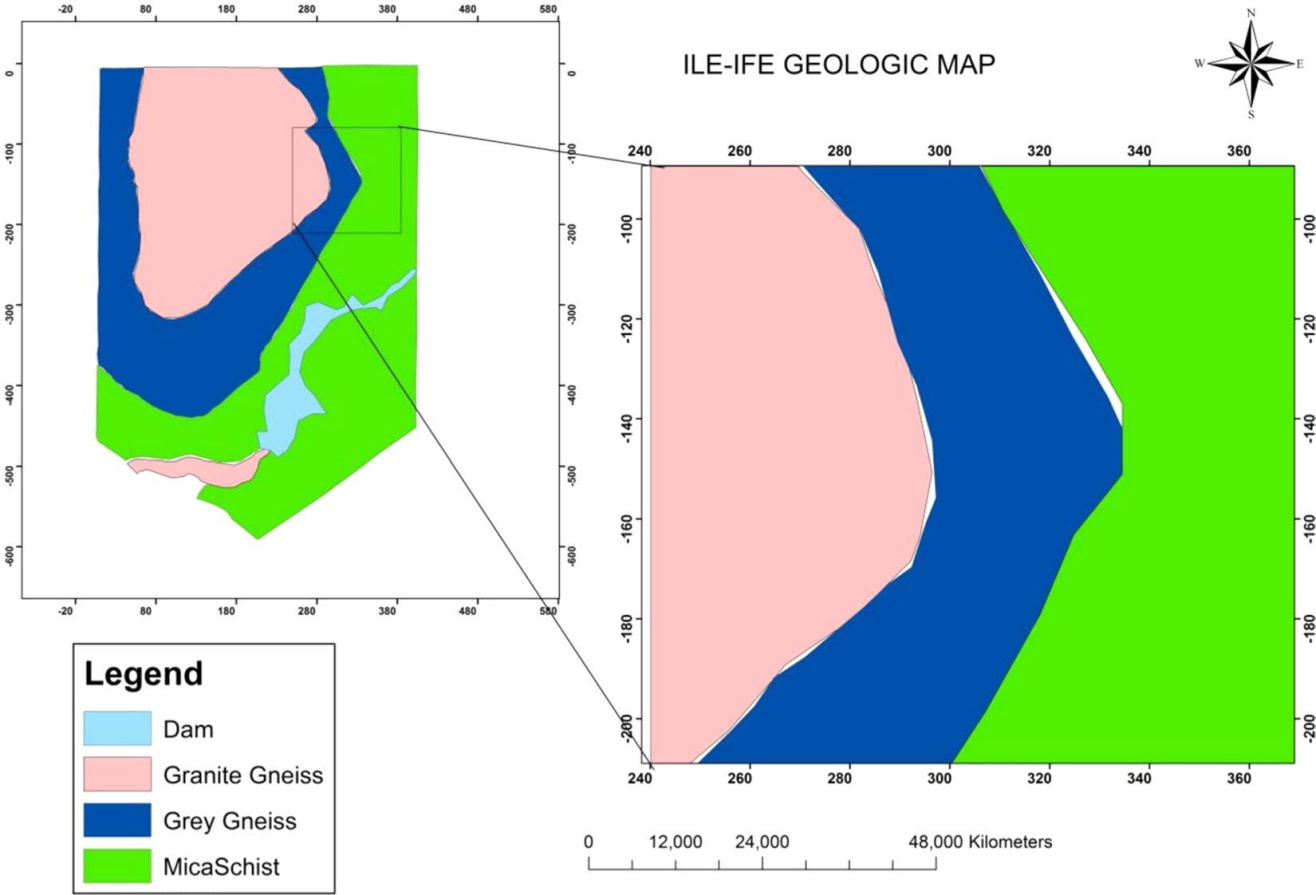
Geological map of the study area showing the three lithological units. The granite gneiss represented with pink colour on the map occupies the western part of the area whereas the grey gneiss lithology represented in blue is sandwiched between grey gneiss and mica schist. The mica schist occupies the eastern part of the area.

The study area lies on the Precambrian basement complex of Nigeria, including the Ife-Ilesha schist belt, unmetamorphosed igneous, and gneiss complex. The lithologic components of the study area have been classified by Rahaman^25^ into gneisses and schists with slight occurrences of ultramafic rocks whose mineral content range from those of the greenschist to amphibolite facies. They grey gneiss, which are the oldest rocks in the study area occurs as low-lying outcrops over half of the entire study site. The granite gneiss appears as inselbergs forming prominent hills with strong foliation of the mineral bands. Granite is sandwiched between grey gneiss indicating a younger age while mica schist occupies the eastern flank of the study area. The landscape is associated with steeply slope gradient ranging from about 6–12%. The bedrock weathered into thick regolith overburden materials that vary from lateritic clay, clayey sand to sand^26^. The geological map of the study area is presented in Fig. 1.

**Figure 2:**
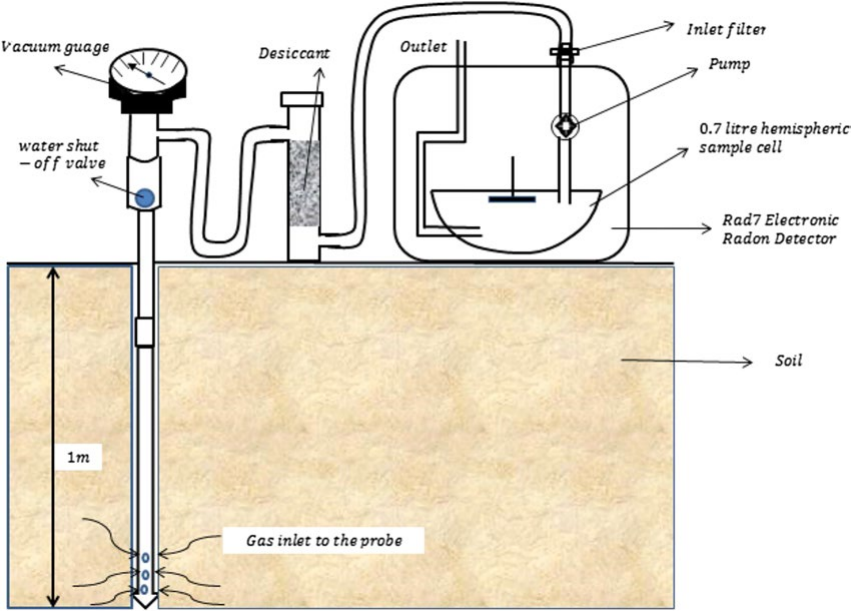
Experimental set-up for radon-in-soil gas measurement (Source: Ajiboye *et al*. 2018). The set-up involves three main components which are (i) the RAD7 electronic radon detector which detects and measures radon concentration, (ii) the desiccant which is a column of CaSo_4_ which eliminates moisture from gas going into the RAD7, and (iii) soil gas probe which in inserted into the soil for radon gas collection.

Soil gas radon measurement was carried out in September and October of 2016. A total of 138 *in-situ* measurements of radon concentration in soil were carried out at different locations spatially distributed within the residential staff quarters of ObafemiAwolowo University, Ile-Ife based on square grid (20 m^2^) sampling method. Each measurement was carried out at the centre of the square grid with the geographical coordinates determined with a geographical positioning system (GPS) manufactured by Garmin. Measurement of the soil gas radon concentration was carried out with a RAD7 electronic radon detector manufactured by Durridge Company, USA. The detector was coupled to a 1 m AMS soil gas probe as described in Fig. 2. The RAD7 detector system is a solid state detector with a hemispheric sampling cell of 0.7 litres. Its detector converts alpha radiation directly into electrical signal. The detector which is an ion-implanted silicon alpha detector is located at the centre of the sampling cell of the RAD7. A high electrostatic potential of about 2000 to 2500 V is applied between the internal wall of the sampling cell and the detector to create a high electric field throughout the volume of the sampling cell. The electric field allows the positive charge particles to be drawn toward the active detector surface. The Rad7 unit pumps soil gas radon into the sampling cell with the aid of an in-built pump.

The measurement was carried out using the following standard protocol. The Rad7 unit was first purged for several minutes by pumping air into the sampling cell. Before the air reaches the cell, it is allowed to pass through a desiccant (drierite) to lower the humidity in the sample cell of the detector. When the relative humidity drops to below 6%, the RAD7 unit is then set to SNIFF protocol. In this protocol, the purging process is continued.However, it allows for the monitoring of the average count per minute (CPM) of the detector. The sniffing protocol is continued until the CPM < 0.5. Once these limits have been achieved, the measurement is then carried out with GRAB protocol.This protocol is programmed such that the detector pump operates for 5 minutes pumping phase wherein it sucks in the soil gas sample. After that, the detector waits for another 5 minutes and then counts for four (5 minutes cycles) to get the accurate results. Since the secular equilibrium between^222^Rn and^218^Po is achieved in less than 20 minutes, the GRAB protocol measurement of 30 minutes measuring time determines the radon concentration in the soil gas sample. At the end of the 30-minute measurement period, the Rad7 automatically prints out a radon concentration result summary. For further analysis of the result, the result is downloadable to a computer system using the Capture Software. The minimum detectable limit of the instrument is 4.0 Bq m^−3^ with a measurement accuracy of + /− 5%. Calibration of the RAD7 unit used in this study was done at the Durridge radon calibration facility at Billerica Massachusetts, United States. The calibration system is compared to a precision of better than 1%, with a secondary standard chamber, which is in turn calibrated by comparison with a National Institute of Standards and Technology (NIST) radon standard supplied through the U.S. Environmental Protection Agency. The calibration system’s accuracy was also checked by making a direct measurement of radon level from the activity and emission of a European standard radon source. The calibration achieves a reproducibility of better than±2%.

### Radon Risk indices of the Building sites of the study area

The radon index generally describes the level of protection a building requires to prevent it against the influx of radon from the underlying soil. Radon index in the present study was determined based on *in situ* measurement of radon concentration in soil and soil permeability of underlying soils to the buildings in the area. These two parameters are useful in determining the radon index of an environment and have been used by several researchers^4,27,28^. This study employed the Barnet *et al*.^27^ model to categorise the soil in the study area into different permeability range as shown in Table 1. The input parameter for radon index assessment is the third quartile of the soil gas radon concentration.

**Table 1:** Classification of radon risk level (Barnet *et al*. 2008).

Radon risk level	Radon Concentration in Soil (kBq m^−3^)		
	Low soil permeability	Medium soil permeability	High soil permeability
Low	${C}_{Rn} < 30$	${C}_{Rn} < 20$	${C}_{Rn} < 10$
Medium	$30\le {C}_{Rn} < 100$	$20\le {C}_{Rn} < 70$	$10\le {C}_{Rn} < 30$
High	${C}_{Rn}\ge 100$	${C}_{Rn}\ge 70$	${C}_{Rn}\ge 30$

*${C}_{Rn}$ is the radon concentration in soil gas.

Based on the previous reconnaissance survey carried out by Ajayi and Adepelumi^26^, the overburden on the three lithologies, granite gneiss, grey gneiss and mica schist are lateritic clay, clayey sand, and sand respectively. Mica schist lithology exhibits medium permeability while grey gneiss and granite gneiss both exhibit low permeability.

### Radon mapping

The Geostatistical analysis was carried out on the spatial soil gas radon concentrations obtained. We applied a variogram estimator in the software package (Surfer 13) and we computed an experimental variogram for the total lithologies observed in the area. The mean radon concentration in the area was predicted applying Kriging to the soil-gas radon concentration values and also considering the nugget effect in the variogram model. Geological information Boesse^29^ was also overlain on the data to observe similarities and trends of the soil gas radon distribution. Soil gas radon data distribution was divided into three groups in accordance to the number of lithologies observed in the area. Soil gas radon distribution variograms were then generated.

## Results and Discussion

**Figure 3:**
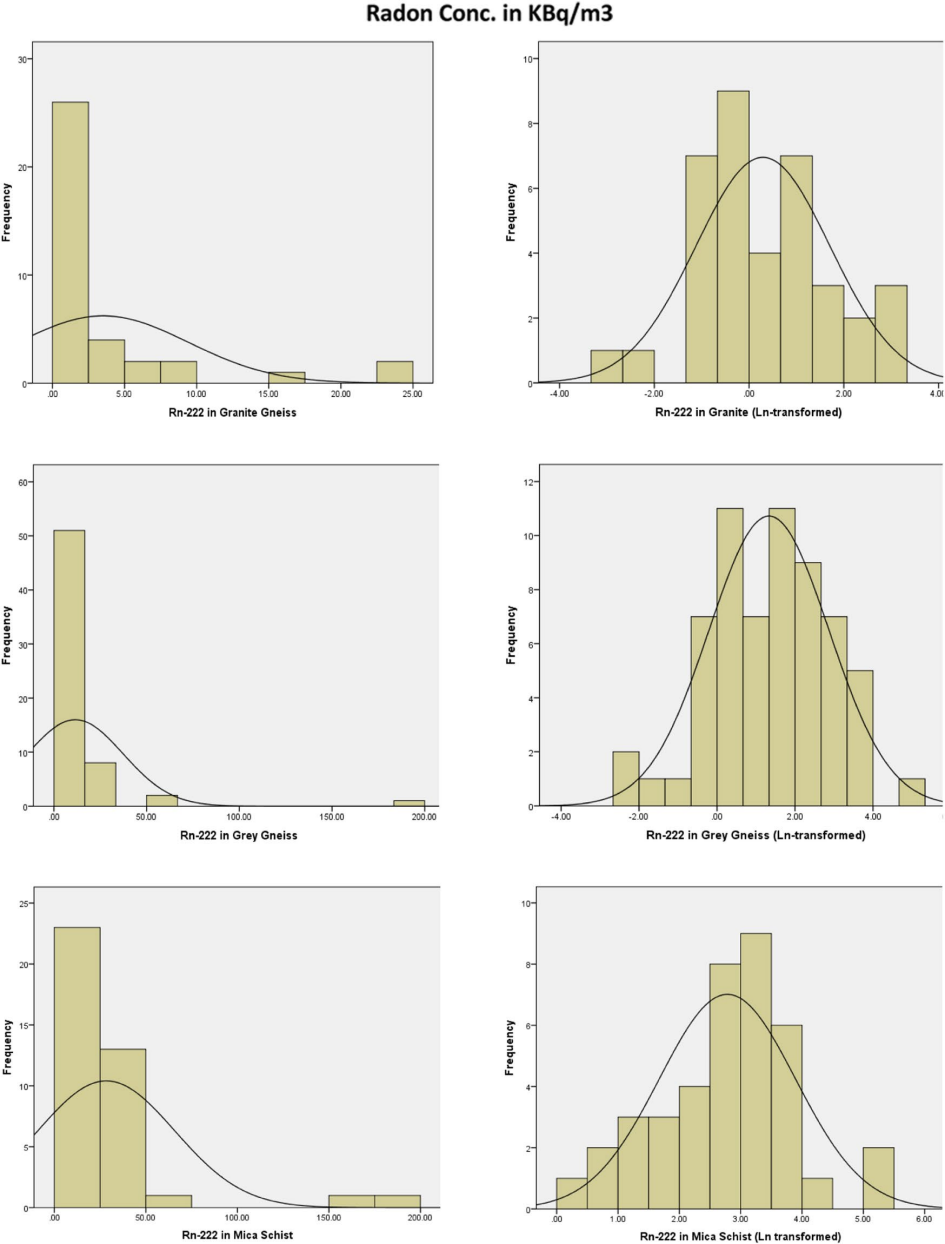
Frequency distribution and Ln-transformed distribution of soil gas radon concentration (kBq/m^3^) in the three Lithologies.

Descriptive statistics of the soil gas radon concentrations obtained in the study area is presented in Table 2. Soil gas radon concentration values obtained across all the sampling locations ranged between 0.04 kBq/m^3^ and 190 kBq/m^3^ with a mean of 14.1 kBq/m^3^. However, in the soil overlying the three lithologies, the concentration values varied from 0.04 to 24.9 kBq/m^3^, 0.10 to 190.0 kBq/m^3^, and 1.55 to 180 kBq/m^3^ with mean values of 3.5 ± 5.9 kBq/m^3^, 11.5 ± 25.7 kBq/m^3^and 28.4 ± 37.4 kBq/m^3^ for granite gneiss, grey gneiss and mica schist respectively. The distributions of the soil gas radon concentration throughout the study area and across the three lithologies of the study area are positively skewed. A skewness value of 4.7 was obtained for the measured soil gas radon concentration across the study area. Whereas across the three lithologies, skewness values of 2.8, 5.8 and 3.3 were obtained for Granite Gneiss, Grey Gneiss and Mica Schist respectively. The frequency distributions of the radon concentrations across the three lithologies with their Ln-transformed frequency distribution are presented in Fig. 3. The Ln-transformed distributions for the three lithologies have peaks at 0.5 kBq/m^3^, 1.3 kBq/m^3^, and 2.7 kBq/m^3^ for granite, grey gneiss and mica schist lithologies respectively. There is statistically significant relationship between soil gas radon concentration and lithology with p < 0.001 (Table 3).

**Table 2:** Descriptive statistics of soil gas radon concentration (kBqm^−3^) across the three lithologies.

Statistic	Granite Gneiss	Grey Gneiss	Mica Schist	Total
N	37	62	39	138
Mean	3.5	11.5	28.4	14.1
Median	1.3	4.0	19	4.2
Minimum	0.04	0.1	1.6	0.04
Maximum	24.9	190.0	180.0	190.0
Std.Dev	5.9	25.8	37.4	28.0
Skewness	2.8	5.8	3.3	4.7
Kurtosis	7.4	38.7	11.5	25.7
Coeff.of Var. (%)	169.0	224.0	131.7	198.6
25^th^ Percentile	0.5	1.2	7.9	1.2
50^th^ Percentile	1.4	4.0	19.0	4.3
75^th^ Percentile	3.2	11.4	32.1	19.0

**Table 3:** Statistical association between Geological units and Radon soil gas concentration.

	Granite Gneiss	Grey Gneiss	Mica Schist	F	P
Mean±SD	3.51 ± 5.92	11.50 ± 25.79	28.37 ± 37.38	8.897	<0.001

The study revealed that soil gas radon concentrations varied across the three lithologies found in the study area in the order: $R{n}_{GraniteGneiss} < R{n}_{GreyGneiss} < R{n}_{MicaSchist}$ with radon concentration in mica schist about eight times greater than that of granite gneiss and about two and half time greater than that of grey gneiss. This finding is in agreement with the study by Lara *et al*.^30^ where radon soil gas in schist exhibited the highest radon concentration in comparison with other lithologies. Although granitic rocks are generally mineralised with uranium – a parent radionuclide of ^222^Rn, however, the overlying overburden contributes to the concentration in soils. The high concentration of radon in the mica schist region in this study is attributable to the low permeability of its overburden. Other similar measurements performed by various researchers showed that the soil gas radon concentration might vary over a wide range depending on weather conditions, climatic factors and soil type^7,30,31^. Also, the radon variation patterns change with time. This may be attributed to the disturbance of site condition by fault movement. The average soil gas radon concentration obtained in this study is lower in comparison with mean radon activity of 38.9 kBq/m^3^ obtained by Ajiboye *et al*.^7^ inEkiti State, Nigeria. The value is however, higher than the maximum value of 2.1 kBq/m^3^ reported for Ibadan, Nigeria by Obed *et al*.^32^.

Using the value of the third quartile, C_A75_ (3.2 kBq/m^3^) for granite gneiss and assuming a medium permeability, the granite gneiss lithology can be classified as a low radon index region (Table 4). Also, the grey gneiss lithology (C_A75_ = 11.37 kBq/m^3^) with medium permeability can be classified as low radon index region. However, for mica schist (C_A75_ = 32.1 kBq/m^3^) with low permeability (as seen in Table 4), the region is classified as medium radon index region.

**Table 4:** Determination of Radon Index.

Lithology/Geologic units	75^th^ Percentile/3^rd^Quartile	Permeability	Category	Radon Index (RI)
Granite gneiss	3.24	Medium permeability	c < 20	Low
Grey gneiss	11.37	Medium permeability	c < 20	Low
Mica schist	32.10	Low permeability	30 ≤ c <100	Medium

The result of the risk criterion based on Swedish classification^30^ is presented in Table 5. According to the Swedish criteria, soils exhibiting radon concentration below 10 kBq m^−3^ are classified as low risk, while those having concentrations within 10 kBq m^−3^ and 50 kBq m^−3^ are classified as normal risk. However, those with concentration exceeding 50 kBq m^−3^ are classified as high risk. On this basis, 65.9% of the sampling locations in this studyfall within the low radon risk areas. Also, 29.7% of the sampling locations fall within normal radon risk areas. However, 4.3% of the sampling areas are high radon risk areas since their soil gas radon concentrations exceed 50 kBq/m^3^.

**Table 5:** Soil gas radon risk classification based on Swedish criteria.

Radon concentration range (kBq/m^3^)	Frequency	Percentage	Category
$$ < 10$$	91	65.9	Low risk
$10\,-\,50$	41	29.7	Normal risk
$$ > 50$$	6	4.3	High risk

**Figure 4:**
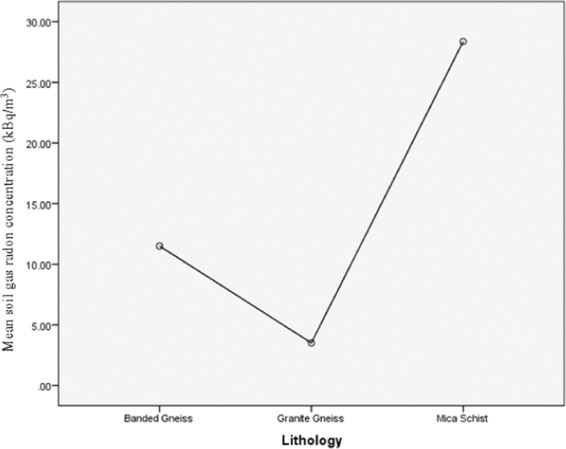
The mean radon soil gas concentration (kBq/m^3^) across the three lithologic unit of the study area.

A variogram which characterises the spatial continuity or roughness of a data set was applied to the individual and total data set. A significant statistical fluctuation was observed in areas underlain by mica schist (Fig. 4). Areas underlain by granite gneiss and grey gneiss had a variogram with similar properties, which indicates a close geologic composition of both lithologies. Also, anisotropy with an angular tolerance of 45 was observed in the entire reading. Spatial correlation is seen, which implies that the underlain rocks in the areas have different properties and response in terms of direction.

**Figure 5:**
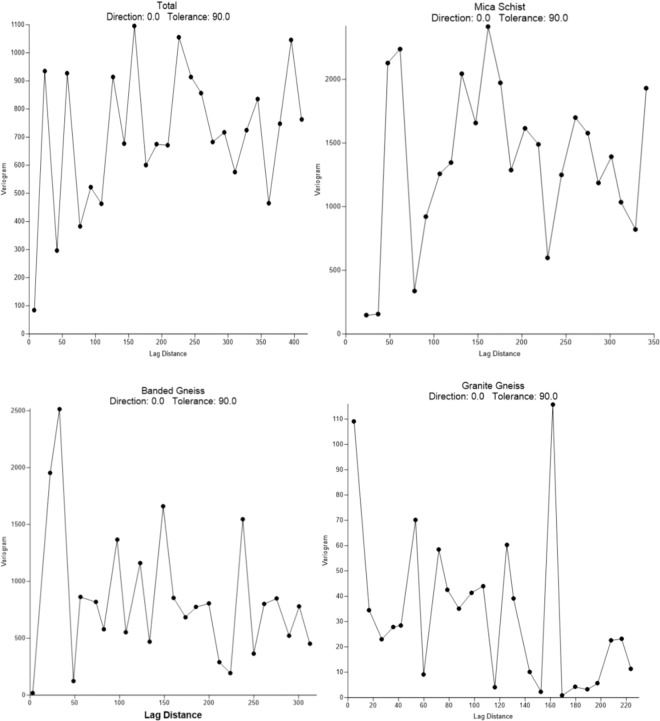
Empirical variograms of Radon concentrations considering the total database and the three geological subgroups: granite gneiss, grey gneiss (Banded gneiss) and mica schist.

The varied level of radon soil gas concentration obtained across the lithologies showed in Fig. 5, could be explained in terms of uranium–radium concentration in the residual mineral fraction of each soil type, the degree of remobilization of members of the radioactive families by weathering and/or post depositional chemical processes, porosity, faulting and advection in case of secondary magma degassing from a deep source. Granites and sheared rocks are generally sources of very high radon^33^. Although, the *in-situ* soil radon concentration over the granite gneiss area in this study appears to be low; it does not necessarily suggest that occupants of building on the area are unexposed to hazard from radon. Low concentration of soil gas radon could however, suggest fast escape/rapid migration of radon to the surface. However, the high concentration of radon concentration found in soil overlying the mica schist region may not be due to the radon produced from the bedrock but rather the low permeability characteristics of this lithology. The mica schist lithology has ability to retain radon gas produced from the bedrock and preventing the escape of the gas from reaching the surface and entering into structures. This however poses an advantage to buildings sited on this lithology; because much of the radon would have decayed before entering into the building structure.

**Figure 6:**
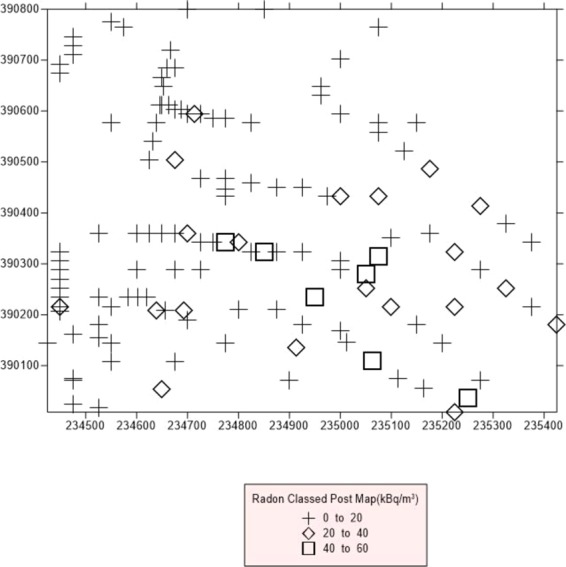
Radon classed post map. The+sign denotes sampled area with soil gas radon concentration (SGRC) between 0 to 20 kBq/m^3^, the ◊ denotes areas of SGRC of 20 and 40 kBq/m^3^ and □ denote areas in the study site with SGRC between 40 and 60 kq/m^3^.

**Figure 7:**
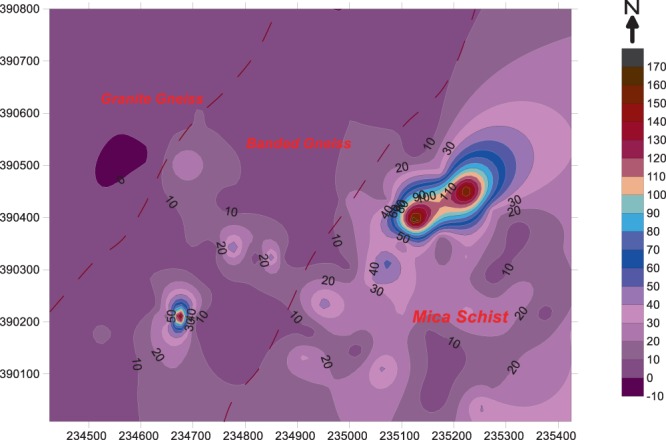
Radon distribution map showing the distribution of radon concentration (kBq/m^3^) across the study area. Most areas within the granite gneiss have radon concentration below 10 kBq/m^3^. Banded gneiss areas exhibit relatively higher concentration, although majority of the areas have radon concentration below 10 kBq/m^3^. Mica schist region have highest concentration ranging between 10 and 170 kBq/m^3^.

A radon classed post map and radon contour map was plotted for the study area (Fig. 6 and 7).The classed post map group the data into discrete classes in addition to XY coordinates (Fig. 6). The radon classed post map revealed the presence of low radon concentration (0–20 kBq/m^3^) in several segments of the entire study area. This signifies the presence of sites with low radon concentrations within the study area and areas with high radon concentrations. The radon contour map, however shows the continuous radon concentration variations in the soil gas radon levels in the study area. As seen from the map, areas underlain by granite gneiss have typical concentrations less than 20 kBq/m^3^. Grey gneiss (banded gneiss) region have varying concentrations between 10 kBq/m^3^ and 90 kBq/m^3^. However, for mica schist region, there is the occurrence of extreme radon concentration values typically 10 kBq/m^3^ and 170 kBq/m^3^.

## Conclusions and Recommendations

A total of 138 soil gas *in-situ* radon measurements were carried out in the residential quarters of ObafemiAwolowo University campus, Ile-Ife. Soil gas radon concentration in the study area exhibit wide variation ranging between 0.04 and 190 kBq/m^3^. The range of radon concentrations obtained across the lithologies of the study area are also distinct. Areas with elevated radon risk have been delineated. The delineated radon map will serve as a useful tool for determination of new building sites in the University and as a guide for future radon remediation plan.

### Ethics approval

Ethical clearance was obtained from UI/UCH ethics review committee of the University of Ibadan, Ibadan.(Protocol number: UI/EC/16/0352).

## Data Availability

The datasets generated/analysed during the current study are available from the corresponding on reasonable request
